# Wingless gene cloning and its role in manipulating the wing dimorphism in the white-backed planthopper, *Sogatella furcifera*

**DOI:** 10.1186/1471-2199-15-20

**Published:** 2014-09-30

**Authors:** Ju-Long Yu, Zhi-Fang An, Xiang-Dong Liu

**Affiliations:** 1 Department of Entomology, Nanjing Agricultural University, Nanjing 210095, China

**Keywords:** Rice planthopper, *Sogatella furcifera*, *Wingless* gene, Wing deformation, Wing length

## Abstract

**Background:**

Wingless gene (*Wg*) plays a fundamental role in regulating the segment polarity and wing imaginal discs of insects. The rice planthoppers have an obvious wing dimorphism, and the long- and short-winged forms exist normally in natural populations. However, the molecular characteristics and functions of *Wg* in rice planthoppers are poorly understood, and the relationship between expression level of *Wg* and wing dimorphism has not been clarified.

**Results:**

In this study, wingless gene (*Wg*) was cloned from three species of rice planthopper, *Sogatella furcifera, Laodelphgax striatellus* and *Nilaparvata lugens*, and its characteristics and role in determining the wing dimorphism of *S. furcifera* were explored. The results showed that only three different amino acid residuals encoded by *Wg* were found between *S. furcifera* and *L. striatellus*, but more than 10 residuals in *N. lugens* were different with *L. striatellus* and *S. furcifera*. The sequences of amino acids encoded by *Wg* showed a high degree of identity between these three species of rice planthopper that belong to the same family, Delphacidae. The macropterous and brachypterous lineages of *S. furcifera* were established by selection experiment. The *Wg* mRNA expression levels in nymphs were significantly higher in the macropterous lineage than in the brachypterous lineage of *S. furcifera*. In macropterous adults, the *Wg* was expressed mainly in wings and legs, and less in body segments. Ingestion of 100 ng/μL double-stranded RNA of *Wg* from second instar nymphs led to a significant decrease of expression level of *Wg* during nymphal stage and of body weight of subsequent adults. Moreover, RNAi of *Wg* resulted in significantly shorter and deformative wings, including shrunken and unfolded wings.

**Conclusion:**

*Wg* has high degree of identity among three species of rice planthopper. *Wg* is involved in the development and growth of wings in *S. furcifera.* Expression level of *Wg* during the nymphal stage manipulates the size and pattern of wings in *S. furcifera*.

## Background

*Wingless/Wnt* signaling pathway is a complicated protein-protein interaction network that regulates important developmental processes, such as cell proliferation, polarity and fate specification [1]. The wingless gene (*Wg*) in *Drosophila* is homologous with the mammalian *Wnt*-1 gene [2], which manipulates embryonic development [3], as well as the limb formation of adults [4]. *Wg* encodes a kind of cysteine-rich secreted protein [5], and the secreted location and concentration gradients mediate the composition of the midgut morphology, development of central nervous system and the formation of imaginal discs of wings, eyes and legs [6-8].

*Wg* protein belongs to a kind of morphogen which acts as a signaling molecule to directly control specific cellular responses [9]. Different concentrations of *Wg* protein lead to different cell reactions. It can stimulate some specific target genes when its concentration reaches a threshold gradient [10,11]. It is well known that *Wg* influences development of *Drosophila* wing imaginal disc [12-14]. *Wg* gene is expressed in a narrow stripe along the dorsal/ventral (D/V) boundary during the development of wing disc of *Drosophila*[15], and it has been considered as one of important signal transduction pathways in the formation of wing pattern [16]. During the formation of wings, *Wg* regulates the expression of downstream target genes through changing its own concentration, and then results in the morphological differentiation of wing imaginal disc cells [9]. When *Wg* gene in *Tribolium castaneum* was knocked down in the late instar of larvae, the wing width of adults decreased [17]. *Wg* gene of *Bombyx mori* exhibited the highest expression level in wing primordium of the fifth instar larvae, and its expression level decreased gradually after pupation. When the expression level of *Wg* was reduced in the fifth instar larvae by RNA interference method, the resulting adults showed a partial or even complete loss of wings [18].

The white-backed planthopper, *Sogatella furcifera* (Hemiptera: Delphacidae), is one of the most devastating pests in rice fields in Asia. It sucks phloem sap of rice and causes a decrease of grain weight [19]. *Sogatella furcifera* has a wing dimorphism which females have either macropterous or brachypterous wings, but males usually are macropterous in China. In their natural environments, the macropterous rice planthoppers can make long-distance migrations to expand their occurrence regions [20]. Although the genetic basis of wing polymorphism in insects is generally not well understood, it has been verified that the wing forms of rice planthopper have a genetic component and are not purely environmentally determined [21]. The titer of juvenile hormone and DNA methylation were also thought to be involved in the determination of wing forms [22-24]. There are some differentially expressed genes between the long- and short-winged brown planthoppers, such as *flightin*, *troponin C4*, *titin* and *myosin heavy chain*[25,26]. However, the molecular characteristics, expression and biological function of *Wg*, an important gene relating to growth and wing imaginal disc development of insects, in rice planthoppers are still not well understood. Moreover, it is unknown whether the *Wg* plays a key role in determining the development of wings and in manipulating the wing dimorphism in rice planthoppers. In the present study, therefore, the full-length cDNAs encoding *Wg* were cloned and characterized from the three common species of rice planthopper, *S. furcifera, Laodelphgax striatellus* and *Nilaparvata lugens*. And then the expression differences of *Wg* between the macropterous and brachypterous lineages of *S. furcifera* which wing forms were selected for more than 20 generations under a constant condition were examined using the quantitative real-time PCR method. Finally, the survival of nymphs, body weight, wing length and wing pattern of adults in the macropterous lineage were measured when the *Wg* expression was knocked down by ingestion of dsRNA of *Wg* in nymphs. This study will illustrate the role of *Wg* in determining the wing dimorphism of rice planthoppers.

## Results

### Selection response of wing forms of *S. furcifera*

**Figure 1:**
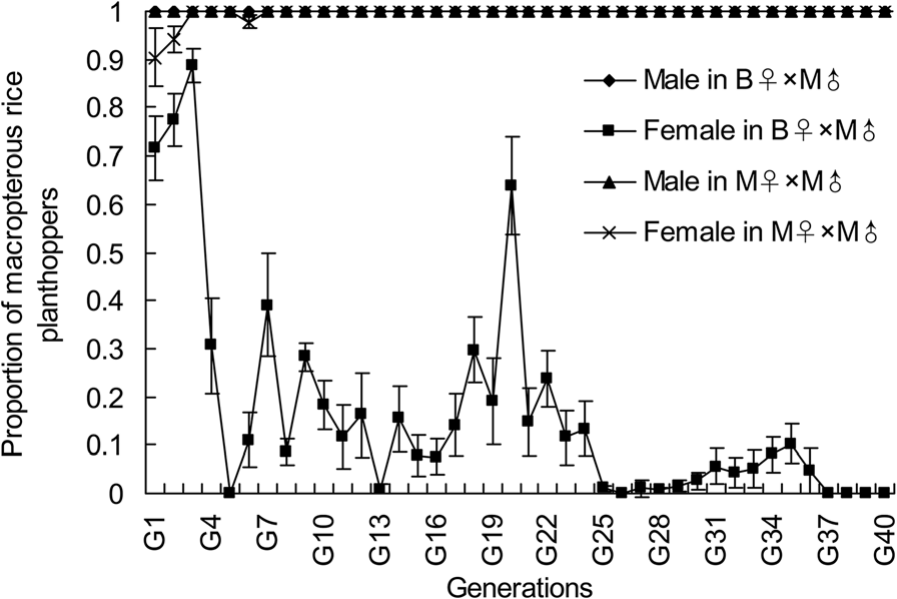
**The proportion of macropterous rice planthoppers in the offspring from the M**♀ × **M**♂ **and B**♀ × **M**♂ **lineages of *Sogatella furcifera *at different generations of selection.**

All the adults from the parents (macropterous female × macropterous male, M♀ × M♂ lineage) were macropterous after seven generations of selection. All the males from the parents (brachypterous female × macropterous male, B♀ × M♂ lineage) were macropterous during all these 40 generations of selection, and more than 95% of the females were brachypterous after 25 generations of selection (Figure 1). The *S. furcifera* had significant selection response in wing forms. The macropterous pure line had been obtained by seven continuous generations of selection from M♀ × M♂ lineage (Figure 1).

### Characteristics of *Wg* from three species of rice planthoppers

**Figure 2:**
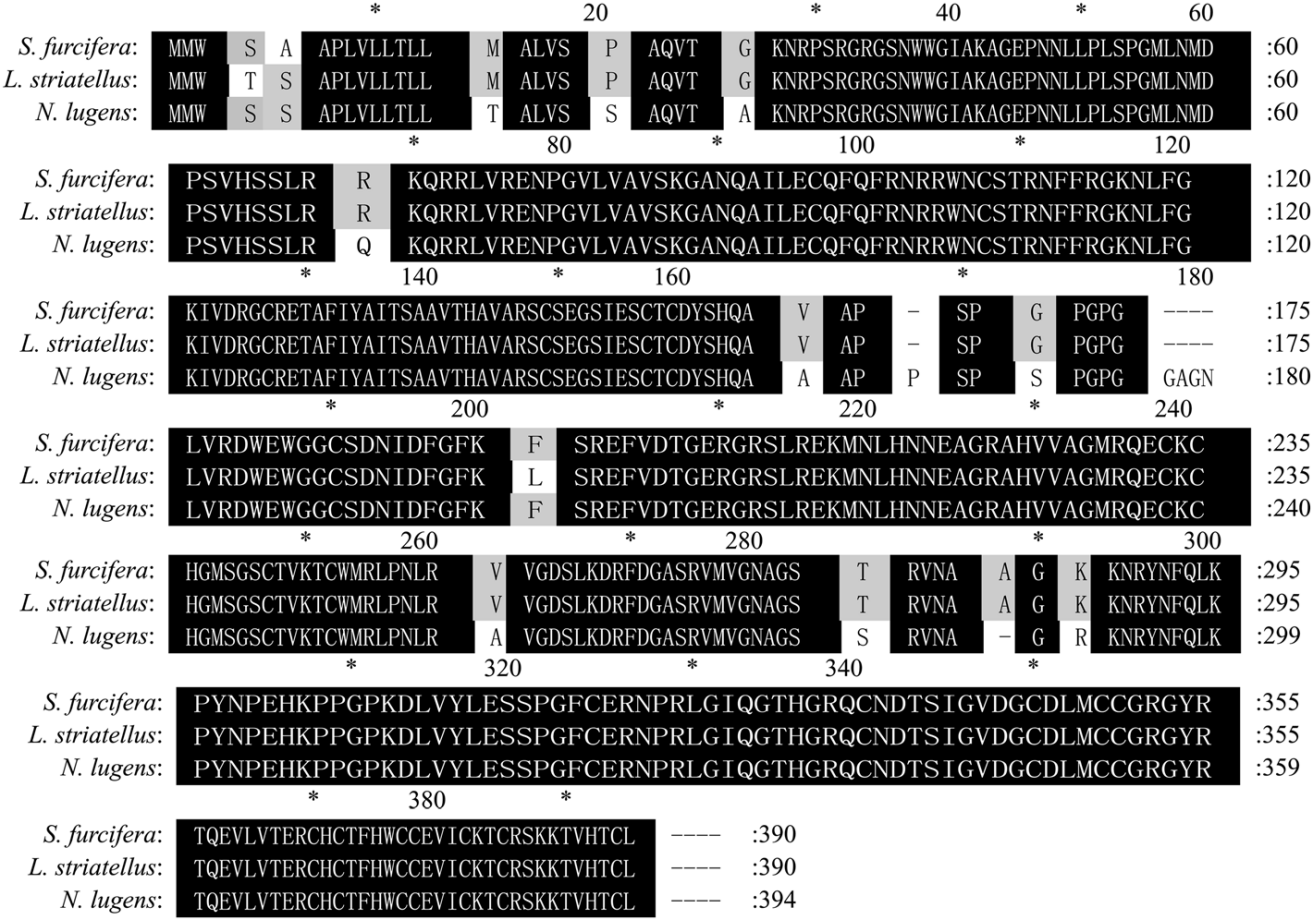
**Alignment of the deduced amino acid sequences encoded by *Wg *from three species of rice planthopper, *Nilaparvata lugens*, *Sogatella furcifera *and *Laodelphax striatellus*.** Amino acids covered by black, grey, and white bars are 100%, 80%, and below 80% identity among the three species of rice planthoppers, respectively.

The full-length cDNA clone encoding *Wg* was isolated from *S. furcifera* and *L. striatellus* by the 3′ and 5′ RACE, as well as the open reading frame (ORF) of *Wg* from *N. lugens.* The cDNA length of *Wg* from *S. furcifera* was 1571 bp which contains 31 bp 5′-untranslated region (UTR), 367 bp 3′-UTR with a consensus polyadenylation sequence and 1173 bp ORF. The deduced protein consisted of 390 amino acid residues. A full-length cDNA of *Wg* from *L. striatellus* was 1443 bp containing 34 bp 5′-UTR,1173 bp ORF, and 236 bp 3′-UTR, which also encoded 390 amino acid residues. The cDNA sequence of *Wg* from *N. lugens* had a complete 1185 bp ORF encoding 394 amino acid residues. The deduced amino acid sequences encoded by *Wg* had high level of identity among the three species of rice planthopper. There were only three different amino acid residues between *S. furcifera* and *L. striatellus. Nilaparvata lugens Wg* had ten and eleven different amino acid residues compared to *S. furcifera* and *L. striatellus*, respectively. The result implied that *Wg* was highly conservative in rice planthoppers (Figure 2).

### Expression of *Wg* in the macropterous and brachypterous lineages of *S. furcifera*

**Figure 3:**
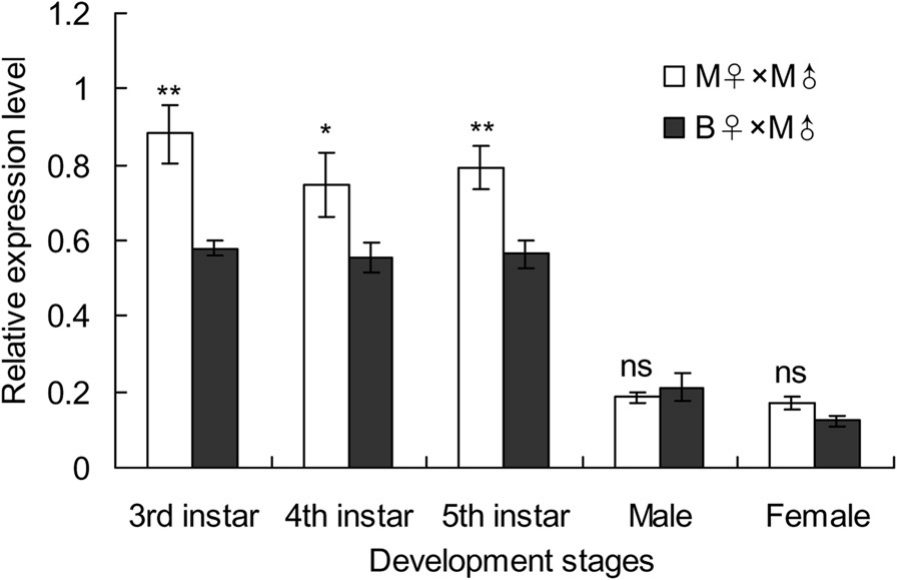
**Relative expression levels of *Wg *mRNA in the macropterous and brachypterous lineages of *Sogatella furcifera*.** Error bars indicate standard error (SE, n = 6). * and ** above the bars indicate significant differences at *P* < 0.05 and 0.01 level between the macropterous and brachypterous lineages, respectively.

Relative expression levels of *Wg* mRNA were significantly higher in nymphs than in adults regardless of the macropterous and brachypterous lineages (for the macropterous lineage: *F*_4, 25_ = 36.876, *P* < 0.0001; for the brachypterous lineage: *F*_4, 25_ = 51.908, *P* < 0.0001). Moreover, the expression levels were significantly higher in the macropterous lineage than in the brachypterous lineage during the 3^rd^ instar (*t* = 4.238, *df* = 5, *P* = 0.0082), 4^th^ instar (*t* = 3.554, *df* = 5, *P* = 0.0163), and 5^th^ instar nymphs (*t* = 5.946, *df* = 5, *P* = 0.0019), whereas there were no significant differences between the two lineages during adults (male: *t* = 0.870, *df* = 5, *P* = 0.4243; female: *t* = 2.018, *df* = 5, *P* = 0.0997, Figure 3).

**Figure 4:**
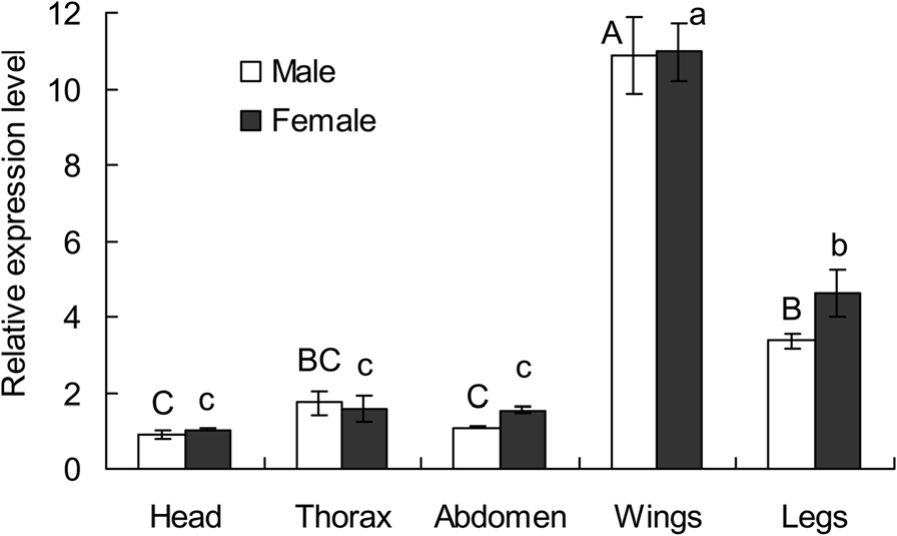
**Relative expression levels of *Wg *mRNA in five parts of the macropterous adults of *Sogatella furcifera*.** Error bars indicate standard error (SE, n = 3). Different capital and lowercase letters above the bars indicate significant differences at *P* < 0.05 level among different parts of male and female adults, respectively.

Relative expression levels of *Wg* mRNA in different parts of the macropterous adults of *S. furcifera,* such as head, thorax, abdomen, wings and legs, showed that *Wg* was expressed mainly in wings and legs, and at much lower levels in body segments: head, thorax, and abdomen (males: *F*_4, 10_ = 73.556, *P* < 0.0001; females: *F*_4, 10_ = 81.891, *P* < 0.0001). Relative expression levels between females and males were not significantly different (Figure 4).

### Survival and body weight of *S. furcifera* after *Wg* RNAi

**Figure 5:**
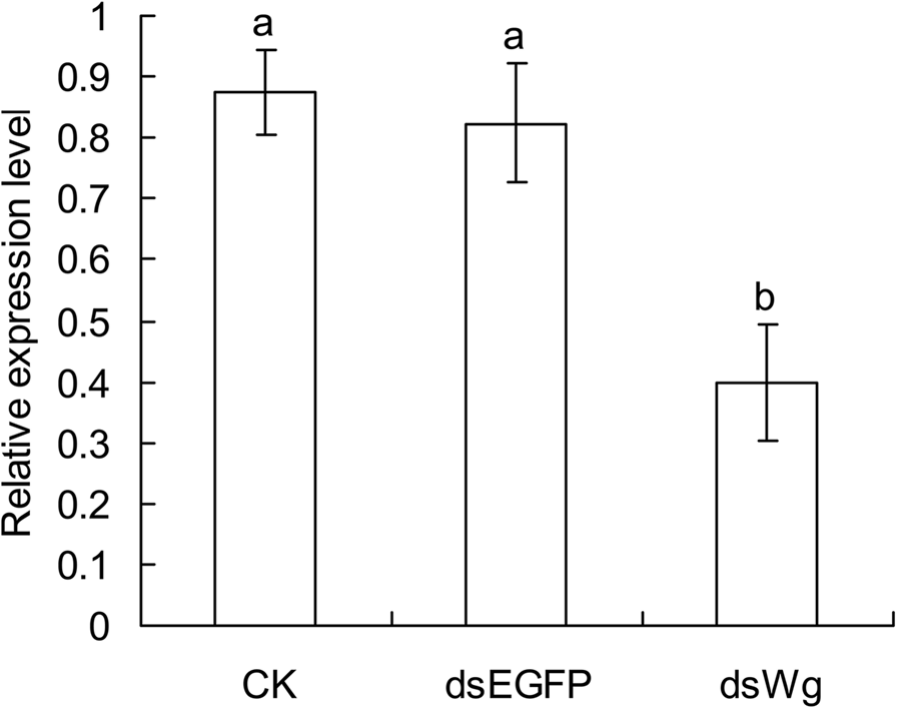
**Relative expression levels of *Wg *mRNA in nymphs of *Sogatella furcifera *feeding on artificial diet with 100 ng/μL dsEGFP and dsWg for seven days.** Error bars indicate standard error (SE, n = 3). Different lowercase letters above the bars indicate significant differences among the *Wg* RNAi and controls at *P* < 0.05 level.

**Figure 6:**
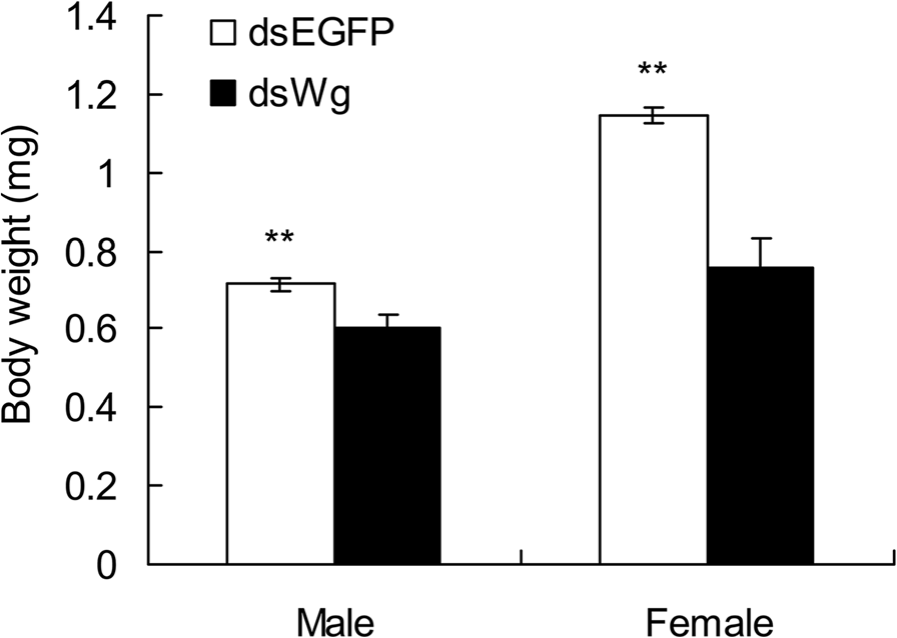
**Body weights of male and female adults when their nymphs were fed with dsEGFP and dsWg.** ** indicates the significant differences between the treatments of dsEGFP and dsWg by a student’s *t* test at *P* < 0.01 level.

The results showed that mortality rates of *S. furcifera* nymphs ingested artificial diet with 100 ng/μL dsWg and dsEGFP were 38.81 ± 3.77% and 30.35 ± 6.54%, respectively, and there were no significant differences (*t* = 1.209, *df* = 8, *P* = 0.261). Mortality of nymphs in the control was 6.41 ± 1.73% which was significantly lower than these dsWg and dsEGFP treated nymphs (*F*_2, 10_ = 11.041, *P* = 0.03). Relative expression levels of *Wg* mRNA in nymphs fed on 100 ng/μL dsWg for seven days significantly decreased (*F*_2, 6_ = 8.836, *P* = 0.018, Figure 5). Body weights of these resulting adults were reduced when they were fed on artificial diet with dsWg during nymphs in comparison with those fed on diet with dsEGFP (males: *t* = 2.934, *P* = 0.008; females: *t* = 5.360, *P* < 0.0001, Figure 6).

### Wings of *S. furcifera* treated by *Wg* RNAi

**Figure 7:**
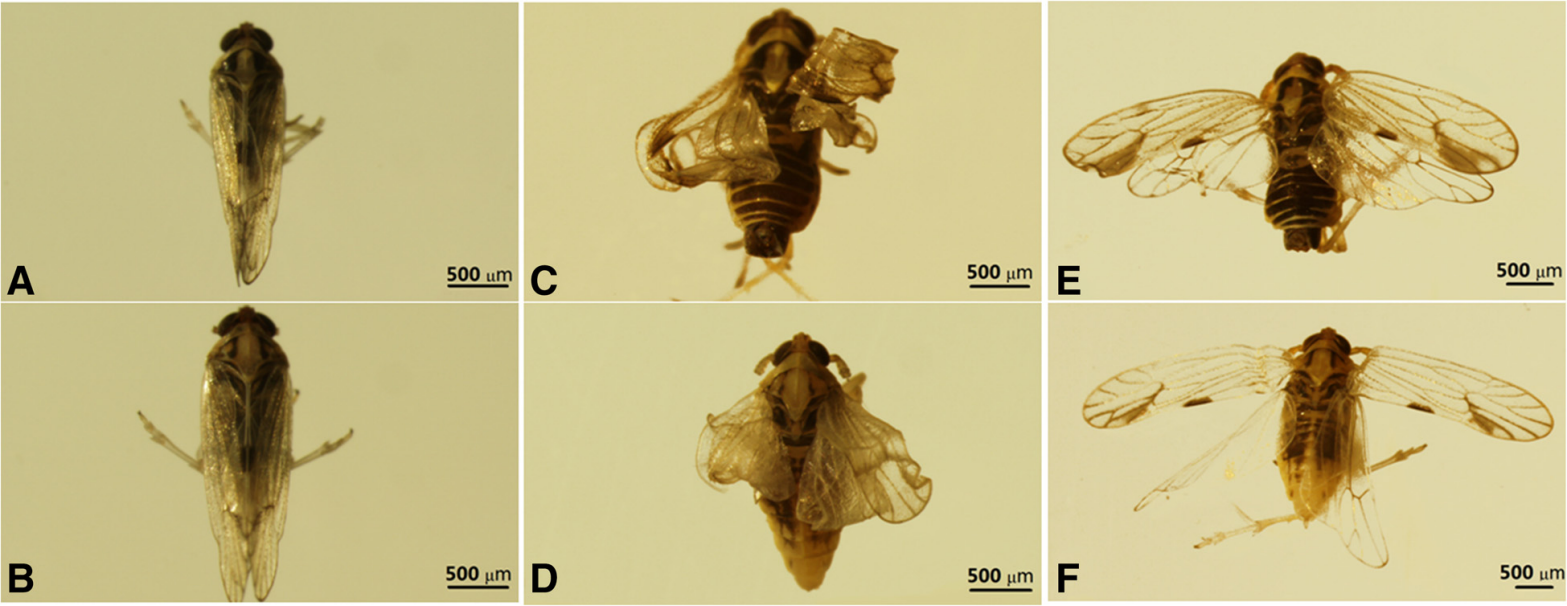
**Wings of adult *Sogatella furcifera. *A** and **B**: nymphs ingested 100 ng/μL dsEGFP. **C-F**: nymphs ingested 100 ng/μL dsWg.

**Figure 8:**
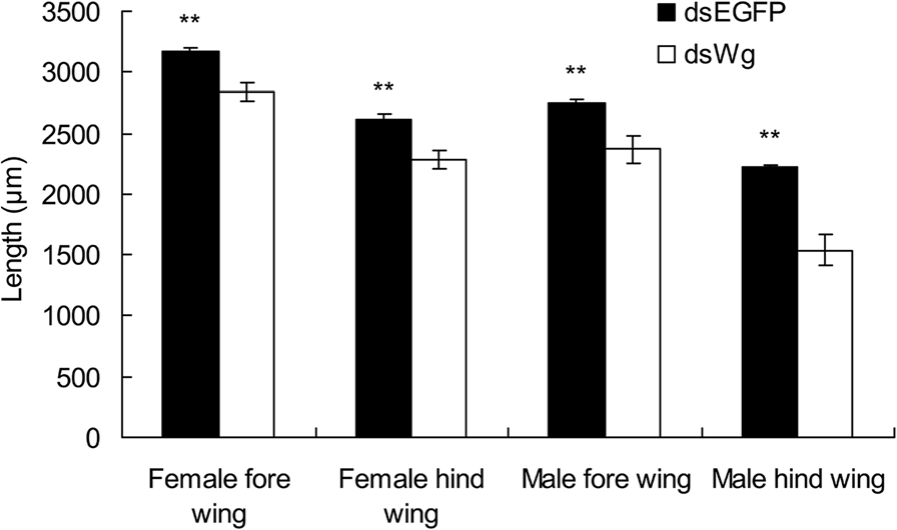
**Lengths of the fore- and hind-wing of *Sogatella furcifera *adults which nymphs were fed on diet with 100 ng/uL dsEGFP and dsWg*.*** ** indicates the significant differences between dsEGFP and dsWg treatments by a student’s *t* test at *P* < 0.01 level.

*Wg* RNAi resulted in seriously deformative wings in *S. furcifera*. The abnormal rates of wings in the blank (CK) and negative controls (dsEGFP) were 1.90% (n = 105) and 1.82% (n = 110), respectively, however, the value increased to 18.55% (n = 124) when the nymphs were fed on diet with dsWg. There were significant differences among dsWg, dsEGFP and CK in the abnormal rates of wings (*x*^2^ = 29.88, df = 2, P < 0.0001). The normal wings of *S. furcifera* were smooth and folded on the back (Figure 7 A and B), but the adults had shrunken (Figure 7C and D) or unfolded wings on the back (Figure 7E and F) when their nymphs were ingested artificial diet with 100 ng/μL dsWg. The wings from nymphs ingested dsWg had obvious patterning defects in the vines and pigmentation, and the background and veins of wings were depigmented (Figure 7). The lengths of fore- and hind-wing from base to tip became significantly shorter when nymphs were fed with 100 ng/μL dsWg than that fed with 100 ng/μL dsEGFP (female fore-wing: *t* = 3.68, *P* < 0.0001; female hind-wing: *t* = 3.68, *P* < 0.0001; male fore-wing: *t* = 4.74, *P* < 0.0001; male hind-wing: *t* = 5.88., *P* < 0.0001, Figure 8). The *Wg* was involved in the determination of wing length and pattern in *S. furcifera.*

## Discussion

The white-backed planthopper is one of insects with wing-dimorphism which adult was either macropterous or brachypterous. It has been reported that the development of wing was controlled by multiple genes [27-30]. The wing pattern genes are mainly involved in determining three kinds of development of insect wings: proximal-distal, dorsal-ventral and anterior-posterior. The *Wg* occupies a key position in the genetic determination system of the development of dorsal-ventral axis, and it directly impacts the expression of downstream genes controlling the wing pattern [9]. In this study, we found the wing forms of *S. furcifera* exhibited a significant selection response, and the macropterous pure line and brachypterous near-pure line were obtained after seven and twenty-five generations of selection, respectively. Additionally, we cloned the full-length cDNA of *Wg* from *S. furcifera* and *L. striatellus*, and the ORF from *N. lugens*. The amino acid residues encoded by *Wg* had more than 99% identity between *S. furcifera* and *L. striatellus*, whereas the value was 94% between *S. furcifera* and *N. lugens.* The *Wg* is highly conservative within the Delphacidae. Among the three species of rice planthopper, *S. furcifera, L. striatellus* and *N. lugens*, the *Wg* from *S. furcifera* had higher similarity with *L. striatellus,* but relatively lower with *N. lugens*. Peng et al. [17] presumed that the genetic basis of wing forms in *S. furcifera* was as similar as *L. striatellus* and both were controlled by two pairs of alleles, one locating on a euchromosome and the other on a sex chromosome, but it was significantly different from *N. lugens* which was controlled by an allele locating on a euchromosome [28]. The variations of *Wg* among these three species of rice planthopper supported the opinion of Peng et al. [17].

In this study, we found that the *Wg* mRNA expressed highly in third, fourth and fifth instar nymphs, but surprisingly, in adult, the expression level of *Wg* mRNA was significantly lower. The nymphs from third to fifth instar are in the process of development and growth of wings, therefore the *Wg* mRNA expression shows a high level. In the adult, the wings are fully formed, so the expression level becomes lower. In pea aphid, an insect that also has the wing dimorphism, there is a trend towards higher *Wg* mRNA expression in the winged morphs than the wingless ones [27]. In the present study, we found that *Wg* mRNA expression levels in the 3^rd^-5^th^ instar nymphs of the macropterous pure line of *S. furcifera* were significantly higher than that in the brachypterous line, but no differences were found in adults between these two lines. Classic *Wingless/Wnt* signaling pathway in *Drosophila* is related to wing formation process, including wing growth and morphogenesis [31]. In our study, we found that the higher expression of *Wg* mRNA in the nymphal stage was beneficial to form the long-winged rice planthoppers. The relative expression of *Wg* mRNA was more abundant in wings than that in the other parts of adult. These results revealed that the *Wg* gene indeed played an important role in the wing development and growth of *S. furcifera*.

Studies had already illustrated that *Wg* was required for wing cell survival, particularly during the rapid growth phase of wing development. The determination of the final wing size of wings in *Drosophila* was only a part of functions of *Wg*[32]. The lower expression level of *Wg* mRNA in the nymphs of the brachypterous *S. furcifera* lineage might be resulted from the requirement of the development of short wings or from other functions of *Wg*.

Nymphs of *S. furcifera* treated with 100 ng/μL ds*Wg* from the second instar for 7 days had abnormal wings as adults, including shrunken and unfolded wings. In the previous studies, it has been found that the mutation of *Wg* caused the loss of wings in adults of *D. melanogaster*[33]. In *Bombyx mori*, the knocking-down of *Wg* expression by dsRNA resulted in partially missing or even absent wings [18]. In *S. furcifera*, we found the wing lengths of the fore- and hind-wing were significantly shortened caused by the ingestion of dsWg. Therefore, the *Wg* was really involved in the development and growth of wings in rice planthoppers. The β-catenin pathway, one of the *Wg* signal pathway, is required for growth and cell fate specification [34]. The decrease of body weight and wing length of adults *S. furcifera* implied that the β-catenin pathway would be disrupted when the nymphs fed on diet with dsWg. The *Wg* performs the function to determine the growth of wings.

The genetic determination of wing morphs in the rice planthoppers is complex. It has been illustrated that the wing form of rice planthoppers is a quantitative trait controlled by many genes [35-37]. We found here that the wing lengths of female and male adults became significantly shorter when the silencing of the *Wg* was performed at nymphal stage, however, the wings were still longer than that of the naturally branchypterous ones. The forewing lengths were 1.2-1.8 mm and 3.2-3.8 mm in the brachypterous and macropterous females of *S. furcifera*, respectively [38]. In this study the wing length from the dsWg nymphs was 2.84 ± 0.07 mm which was an intermediate wing form. We also found that the relative expression level of *Wg* mRNA in third instar nymphs of the brachypterous lineage was as about 66 percent as the macropterous lineage, and wing length of brachypterous female in natural field was as 47 percent as the macropterous one [38]. However, when the *Wg* mRNA expression level in the macropterous lineage was interfered 50 percent by dsWg in nymphs, the wing length of female was only shortened 10 percent. The expression levels of *Wg* mRNA in nymphs were not linearly related with the wing lengths of rice planthoppers. We presumed that the wing forms of rice planthoppers might be a threshold response to *Wg*. When the expression level of *Wg* was less than a lower threshold gradient, the adults would be short-winged. Otherwise, when it was more than a higher threshold, the adults would be long-winged. Fortunately, we have attained an adult which wings only covered its abdomen when the nymph was fed with a higher concentration 400 ng/uL of dsWg. Of course, this comprehensive process of *Wg* manipulating the wing size and pattern of rice planthoppers still needs the further study.

## Conclusions

The wingless gene has a high level of identity among the three species of rice planthopper, and was strongly related to the development and growth of wings. The expression levels of *Wg* mRNA were significantly higher in the macropterous lineage than in the brachypterous lineage of *S. furcifera.* Interference of *Wg* resulted in the shorter and abnormal wings in *S. furcifera*.

## Methods

### Experimental insects

Three species of rice planthoppers, *S. furcifera*, *N. lugens* and *L. striatellus*, were originally collected from rice fields in Nanjing, Jiangsu province, China, and then reared in laboratory at 25 ± 1°C, relative humidity 75 ± 10%, and 14 h light/10 h dark photoperiod using the rice seedlings (var. Wuyunjing 7).

### Selection of the macropterous and brachypterous lineages of *S. furcifera*

Due to shortage of the brachypterous males in *S. furcifera* in Jiangsu province, only two mating combinations: macropterous male × macropterous female (M♂ × M♀, called the macropterous lineage) and macropterous male × brachypterous female (M♂ × B♀, called the brachypterous lineage) were established, and reared under a constant condition (25 ± 1°C, RH 75 ± 10%, and 14 h light/10 h dark). In their offspring generations, only the females and males with the same wing form as their parents were chosen to mate and propagate. The detail method for selection experiment of wing forms was described by Peng et al. [28]. Continuous 40 generations of selection were performed, and the macropterous and brachypterous lineages were used in the following experiments.

### Cloning of the wingless gene cDNA from three species of rice planthopper

Total RNA was isolated from the homogenization of 10 nymphs of rice planthopper (two individuals from each instar) and four adults (two macropterous males, and one macropterus and one brachypterous female) with Trizol (Invitrogen Co., USA). Total RNA 1 μg was used to synthesize the templates for cloning the *Wg* cDNA using the BD SMART™ RACE cDNA Amplification Kit (Clontech). The PCR primers were SfGSP1-5′: 5′-AGAAGCCAGGCGACGACTCCAGGTAG-3′, SfNGSP1-5′: 5′-CGTCGAACCTGTCCTTGAGGCTGTC-3′, SfGSP2-3′: 5′-ACTTTGGATTCAAGTTCTCGCGGGAG-3′, and SfNGSP2-3′: 5′-AAGCCCTACAACCCGGAGCACAAGC-3′ for *S. furcifera*, and LsGSP3-5′:5′-CGTCGAACCTGTCCTTGAGGCTGTC-3′, LsNGSP3-5′:5′-AAGCCCTACAACCCGGAGCACAAGC-3′, LsGSP4-3′:5′-CACAACACATCAGGTCGCAGCCGT-3′, and LsNGSP4-3′:5′-TTGTGCTCCGGGTTGTAGGGCTTCAG-3′ for *L. striatellus*. PCR reaction system 25 μL was used which contained 1 μL template, 2.5 μL 10 × *Taq* buffer (Mg^2+^ free), 2 μL MgCl_2_ (25 mM), 2 μL dNTP mixture (2.5 mM/each), 1 μL forward and 1 μL reverse primers, 0.25 μL *Taq* polymerase (Takara Bio.) and 15.25 μL double distilled H_2_O. PCR was preceded by denaturation at 94°C for 2 min, followed by 5 cycles at 94°C for 30 s and 72°C for 5 min, and by another 5 cycles at 94°C for 30 s, 70°C for 30s, and 72°C for 4 min, and then by 25 cycles at 94°C for 30 s, 68°C for 30 s, 72°C for 4 min and finishing with chain extension at 72°C for 10 min. The amplified product was separated by 1.5% agarose gel, and the target product was recycled and purified with the Wizard DNA Gel Extraction Kit (Unigenes Promega, Madison, Wis., USA), cloned into the EASY-T3 vector (TransGen Biotech, Beijing, China), and transformed into the DH5α competent cells. Positive clones were chosen to sequence. According to the sequences of *Wg* in *S. furcifera* and *L. striatellus*, the end to end primers *sfwg*-F2 5′-GATGGCAGCGCAATGATG-3′ and *Sfwg*-R2 5′-CGCTGTGGGTTTGGGTAA-3′ for *S. furcifera, Lswg*-F2 5′-AGTACAAGCCTGGTGATGG-3′ and *Lswg*-R2 5′-GGCAATAAAGTGAATTGAATAA-3′ for *L. striatellus* were designed to check the full-length of *Wg*. Unfortunately, We did not clone the full-length of *Wg* from *N. lugens*, and only cloned its ORF used the end to end primers for *S. furcifera.* The ORF sequences of *Wg* gene in the three species of rice planthopper were found using the ORF Finder online service in NCBI (http://www.ncbi.nlm.nih.gov/gorf/orfig.cgi).

### Expression level of *Wg* mRNA in the macropterous and brachypterous lineages of *S. furcifera*

The 3rd, 4th and 5th instar nymphs and adults from the 29^th^ generation of the macropterous lineage and the 30^th^ generation of the brachypterous lineage of *S. furcifera* were collected and frozen in 300 μL Trizol for 24 hours at -70°C. The total RNA from six individuals was isolated using Trizol. The samples were grinded well by mixing some quartz rocks. The total RNA was adjusted to 1 μg/μL with DEPC-treated H_2_O, and 2 μg RNA was used for RT-PCR in the 20 μL reaction mixture using the One Step SYBR PrimerScript RT-PCR Kit (Takara) to synthesize the first-strand cDNA. The real-time qPCR was performed on an ABI 7500 Real-Time PCR System (Applied Biosystems, Foster City, CA, USA) to quantify the expression levels of *Wg*. The first-strand cDNA (2 μL) diluted 50-fold was used as templates in each 20 μL reaction mixture. The qRT-PCR was performed at 95°C for 30 s, and then for 40 cycles at 95°C for 5 s and 60°C for 34 s. The ribosomal 18 s rRNA was used as an internal control. Relative expression level of *Wg* mRNA was computed based on the internal control gene using the 2^-△△Ct^ method. The special primer sets for quantifying *Wg* in *S. furcifera* were the sense primer (*SFwg*-F) 5′-TCGAATGCCAATTCCAGTTTAG -3′ and the antisense primer (*SFwg*-R) 5′-CCCCTATCCACAATCTTTCCA -3′. And in the internal control, the primers for *S. furcifera* 18S rRNA gene were 18S rRNA-F 5′-ACAAGTATCAATTGGAGGGCAAGTCTGG-3′ and 18S rRNA-R 5′-ATGCACACAGTATACAGGCGTGACAAG -3′. The expression levels of *Wg* mRNA in head, thorax, abdomen, wings and legs were also detected in the macropterous lineage of *S. furcifera* which was selected for 37 generations. For each sample, we carried out six biological replicates.

### Synthesis of the double stranded RNA (dsRNA)

A 246 bp of nucleotide sequence of *S. furcifera Wg* was cloned using the sense primer ds*Wg* F: 5′- GCTGCCCAACCTGCGCGT-3′ and antisense primer ds*Wg* R: 5′- CCGTGCGTGCCCTGGATG-3′ designed by the coding region sequence of *Wg*, and inserted into the EASY-T3 vector (Trans Gen Biotech, Beijing, China) to confirm the sequences. The plasmid containing the specific nucleotide sequences was extracted by the MinBEST plasmid Purification Kit (Takara). The diluted plasmid was used as templates for amplification of the target sequence (dsWg) by PCR. The sense primer was T7 *Wg* F: 5′-TAATACGACTCACTATAGGG GCTGCCCAACCTGCGCGT-3′ and the antisense primer was T7 *Wg* R: 5′- TAATACGACTCACTATAGGG CCGTGCGTGCCCTGGATG-3′. The PCR procedures for synthesizing dsWg were performed at 94°C for 2 min followed by 35 cycles at 94°C for 30 s, 55°C for 20 s and 72°C for 20 s, and the last cycle was followed by final extension at 72°C for 5 min. The T7 RiboMAX™ Express RNAi System (Promega USA) was used to produce the specific dsRNA. The dsRNA synthesized here was washed twice using 70% ethanol, dried and suspended into an appropriate amount of the nuclease-free water, and then quantified by the Nano Drop spectrophotometer (Thermo Scientific, Wilmington, DE, USA) at 260 nm. The quality and size of the dsRNA products were verified by 1.5% agarose gel electrophoresis. Enhanced green fluorescent protein gene dsRNA (dsEGFP) was used as the negative control. The sense primer was T7 EGFP-R 5′-TAATACGACTCACTATAGGGAAGTTCAGCGTGTCCG-3′ and the antisense primer was T7 EGFP-F 5′-TAATACGACTCACTATAGGGCACCTTGATGCCGTTC-3′ for synthesizing the dsEGFP. The synthesized dsRNA was stored at -70°C.

### Nymph RNAi

To determine the function of *Wg* in *S. furcifera*, the second instar nymphs were fed on artificial diet with the dsWg [39-41]. Groups of 30 second-instar nymphs were collected from the 37th generation selection of the macropterous lineage of *S. furcifera*, and placed into a feeding chamber constructed from a 3 cm diameter and 12 cm height cylindrical glass tube with one end covered by insect-proof nylon mesh net (48-micron) and the other end covered by two-layer of Parafilm sandwiched together [42]. Seventy-microliter liquid diets containing 100 ng/ul dsWg were put into the two layers. The nymphs feeding on artificial diet containing 100 ng/ul dsEGFP and water were the negative and blank control, respectively. The artificial diet was replaced daily. After seven days on the diet, the nymphs were counted and collected to examine the expression levels of *Wg* mRNA by qPCR method. When the nymphs grew into adults, their survival, wing forms, wing lengths, and body weights were measured. Curled wings were unfolded by dipping in absolute ethanol for 48 hours before measurement. The lengths of wings were measured from the base to tip under a stereomicroscope using the CellSens V 1.5 system (Olympus ®). The dsWg and dsEGFP treatments were repeated at least three times.

### Data analysis

*Wg* protein sequences in three species of rice planthopper were aligned in a multiple sequence alignment using CLUSTAL X [43]. The differences between two means, such as the relative expression levels of *Wg* mRNA between the macropterous and brachypterous lineages, were compared by a student’s *t* test. The expression levels of *Wg* mRNA, body weight and wing length among different tissues or RNAi treatments were analyzed by the ANOVA followed by the Tukey test. The proportions of adults with abnormal wings among RNAi treatments by dsWg and dsEGFP and the control were compared by the Chi square analysis using the Crosstabs method. All these statistic analyses were performed using SPSS 13.0 software (SPSS, Chicago, IL, USA).

## Abbreviations

*Wg*: Wingless gene; *EGFP*: Enhanced green fluorescent potein; PCR: Polymerase chain reaction; RT-PCR: Reverse transcriptase PCR; qRT-PCR: Quantitative real-time PCR; cDNA: Complementary DNA; RACE: Rapid-amplification of cDNA ends; dsRNA: Double-stranded RNA; RNAi: RNA interference; ORF: Open reading frame; UTR: Untranslated region; SE: Standard error; ANOVA: Analysis of variance.

## Competing interests

The authors declare that they have no competing interests.

## Authors’ contributions

JLY and ZFA carried out the experiments. XDL and JLY participated in the data analysis and drafted the manuscript. XDL conceived and supervised the research. All authors read and approved the final manuscript.
